# Retraction: *Eucommia ulmoides* Cortex, Geniposide and Aucubin Regulate Lipotoxicity through the Inhibition of Lysosomal BAX

**DOI:** 10.1371/journal.pone.0332764

**Published:** 2025-09-22

**Authors:** The PLOS One Editors

After this article [1] was published, concerns were raised regarding results presented in Figs 1-2, 4-5, and 7.

Specifically:

- The following panels appear to partially overlap:
  ◦ Fig 2E Cytosol Cathepsin B lanes 2-7 and Fig 7D Caspase-3 panel lanes 2-7
  ◦ Fig 4B Bafi and Fig 4B Pal+EUE+Bafi
- There appear to be vertical discontinuities in the following panels:
  ◦ Fig 1C BAX, tBID, and LAMP-1 between lanes 5 and 6
  ◦ Fig 2E Cytosol Cathepsin B between lanes 7 and 8
  ◦ Fig 5H Lysosome Tubulin and Cytosol BAX between lanes 5 and 6
- The Fig 2A Con and Pal lower panels in [1] appear similar to the Fig 2A Con and Pal panels in [2, retracted in 3].

Corresponding author HJC stated that the original data for Fig 7D Caspase-3 and Fig 4B are no longer available. These concerns cannot be resolved in the absence of the original underlying data.

Corresponding author HJC provided underlying blot data for Fig 1C, Fig 2E, Fig 5H Lysosome BAX, LAMP-1 and Tubulin, and Fig 5H Cytosol Cathepsin B, Tubulin, and Lamp-1 panels. Upon editorial review, these underlying data were insufficient to resolve the above blot concerns, and raised further concerns for the validity and reliability of the published results in these figures.

Regarding the Fig 2A Con and Pal panels in [1], corresponding author HJC stated that these images in [1] and the Fig 2A Con and Pal images in [2, retracted in 3] are the same, as the two studies were part of a broader research project and the Con and Pal conditions were shared across experiments. The *PLOS One* Editors note that the Fig 2A caption in [1] and the Fig 2A caption in [2, retracted in 3] describe different concentrations of palmitate, therefore PLOS remains concerned for the reliability of Fig 2A in [1].

In light of the above concerns that question the reliability and validity of the reported results, the *PLOS One* Editors retract this article.

HJC did not agree with the retraction and stands by the article’s findings. GHL, MRL, HYL, SHK, HKK, and HRK either did not respond directly or could not be reached.
